# Does physical ill-health increase the risk of suicide? A census-based follow-up study of over 1 million people

**DOI:** 10.1017/S2045796020000529

**Published:** 2020-07-08

**Authors:** I. N. Onyeka, A. Maguire, E. Ross, D. O'Reilly

**Affiliations:** 1 Centre for Public Health, Queen's University Belfast, Royal Hospitals Site, Grosvenor Road, Belfast BT12 6BA, UK; 2 Administrative Data Research Centre Northern Ireland, Centre for Public Health, Queen's University Belfast, Royal Hospitals Site, Grosvenor Road, Belfast BT12 6BA, UK

**Keywords:** Data linkage, health, mortality, population, suicide

## Abstract

**Aims:**

Mental ill-health is a known risk factor for suicide mortality. However, the relationship between physical ill-health and suicide is less clear. This study examined the relationship between different aspects of physical ill-health and the risk of suicide death.

**Methods:**

Data for 1 196 364 adults (aged 18 years and over) were identified from the 2011 Northern Ireland Census and linked to death registrations until the end of 2015. Multivariate logistic regression was used to construct models to test associations with likelihood ratio tests for interactions.

**Results:**

Over one in eight individuals (13.7%) reported multimorbidity (⩾2 physical health conditions) and one in four (25.4%) identified having limitation of daily activities. During follow-up, 51 672 individuals died; 877 due to suicide. The gradient in suicide risk by number of physical conditions disappeared following adjustment for activity limitation. Individuals with a lot of activity limitation were over three times more likely to die by suicide (OR = 3.13, 95% CI 2.50–3.93) compared to those with no limitations though this was reduced to OR = 1.72 (95% CI 1.35–2.20) with adjustment for poor mental health. The relationship between activity limitation and suicide was most pronounced at younger ages (18–34 years).

**Conclusions:**

This study suggests that it is the effect that physical illness has on a person's life, in terms of disruption to daily activity, rather than the number of conditions that predicts suicide risk, especially at younger ages. Improved awareness and better management of mental wellbeing of individuals with physical health conditions may help to reduce suicides, especially in younger people.

## Introduction

Mental ill-health is an established risk factor for suicide, demonstrating a consistent, strong, independent and dose-response relationship over a range of studies and populations (Cavanagh *et al*., 2003; Arsenault-Lapierre *et al*., 2004; Bell *et al*., 2015). However, the relationship between poor physical health and suicide risk is unclear. This is important not only in increasing our understanding of suicide behaviour in general but given our ageing population the number of people with multiple health conditions is increasing globally (Barnett *et al*., 2012; World Health Organization [WHO], 2016).

It is known that there is a close relationship between physical and mental ill-health. Individuals with multiple physical health conditions tend to have a lower quality of life (Fortin *et al*., 2004); a higher likelihood of mental health disorder (Barnett *et al*., 2012) and cross-effects exist whereby past mental health has an effect on present physical health and vice versa (Ohrnberger *et al*., 2017). Type and number of physical health conditions have been suggested to be associated with an increased risk of suicidal ideation and suicide attempts (Scott *et al*., 2010; Thompson *et al*., 2014). However, these studies are based on small, non-representative samples of the population, including a sample limited to armed forces veterans (Scott *et al*., 2010; Thompson *et al*., 2014). Studies exploring physical health conditions co-occurring with mental health conditions have similarly suggested an increased likelihood of suicidal thoughts and behaviours but not above the independent risk of mental ill-health alone (Kavalidou *et al*., 2017, 2019). However, this may not translate into an actual risk of suicide as the epidemiology of suicide ideators or attempters differs from that of suicide completers (O'Connor and Kirtley, 2018).

Evidence on the relationship between physical health and death by suicide is mixed. A large multi-state study in the USA suggested that a range of physical health conditions such as; back pain, traumatic brain injury, cancer, congestive heart failure, chronic obstructive pulmonary disorder (COPD), HIV/AIDS, migraine, renal disease and sleep disorders, are associated with an increased risk of suicide and multimorbidity was associated with a 2-fold increased risk of death by suicide (Ahmedani *et al*., 2017). However, this study had limited individual-level socio-demographic information, was limited to those within well-resourced health systems and was not representative of the population. Two recent systematic reviews and meta-analyses demonstrating a higher likelihood of suicide among individuals with COPD and cancer have corroborated some of Ahmedani *et al*.'s findings (Amiri and Behnezhad, 2019; Sampaio *et al*., 2019). A study using a linked dataset of Hospital Episode Statistics and mortality records for England found an association between some physical illnesses such as epilepsy and asthma and risk of suicide (Singhal *et al*., 2014). However, being a clinical sample, results from this study explored hospitalisation from physical illness and suicide risk which may reflect exacerbation of physical illness more than the overall effect of living with physical ill-health on suicide risk. Another USA study found an association between restless leg syndrome and the risk of suicide or self-harm, but due to small numbers could not distinguish between these two outcomes (Zhuang *et al*., 2019). A study based on the Nurses' Health study also found an association between multimorbidity and suicide risk, but again was limited in its representativeness (Wei and Mukamal, 2019). There is clear evidence of a need for more population-wide studies to explore the association between physical health and suicide risk.

A large insurance register-based study in the USA found that having any of nine specific physical illnesses was not associated with suicide risk (Miller *et al*., 2008). A Danish register-based population-wide study found that an association between physical health and suicide attempt was attenuated by adjustment for socioeconomic status suggesting it is poverty more than physical health status that is predictive of suicidal behaviour (Christiansen and Stenager, 2012). In addition, a USA study found that physical health condition was not an independent risk factor for suicide but functional limitation was (Kaplan *et al*., 2007). In their study, Kaplan *et al*. (2007) assessed the existence of functional limitation by asking a question to respondents with a physical health problem: ‘Does any health problem now keep you from working at a job or business, keeping house, going to school, or something else?’. This question wording and binary response failed to capture the chronicity or duration of the activity limitation and the degree of limitation which may both be important for suicide risk. A psychological autopsy study conducted among older adults aged 60 years and over in rural China found that physical illness was not an independent risk factor for suicide but instead severely impaired capability for daily living activities was (Cao *et al*., 2019). This Chinese study assessed activity limitation using a scale and categorised the total score into normal, mildly impaired and severely impaired. However, the study was limited in its representativeness of the various age groups within a community and the methodological challenges of collecting data using proxies.

One further unanswered question is whether the relationship between physical ill-health and suicide risk is modified by age. The prevalence of physical morbidity and also multimorbidity increases with age (Barnett *et al*., 2012), and suicide triggered by physical health problems are commonly reported in older adults (Conejero *et al*., 2018). A recent study highlighted a rising trend in the rate of assisted suicide in this age group (Steck *et al*., 2018) and older adults might be differentially affected by suicide associated with physical ill-health relative to younger people. However, we know that suicide is reported in higher numbers in young people (O'Neill *et al*., 2018).

This study will link population-wide data on physical health, activity limitation and mortality to examine the association between physical health and suicide; specifically, to determine which aspects of physical health (number of conditions or the effects of these conditions on daily activities) is more important for suicide risk, and to determine if the relationship between physical health and suicide varies according to age.

## Methods

### Data sources

**Fig. 1.**
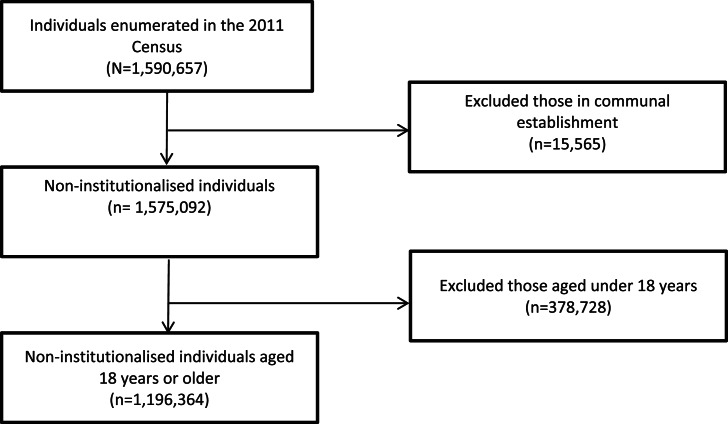
Flowchart showing the sample selection process. Participants were from the 2011 Census records.

A population-wide cohort of all 1 196 364 non-institutional dwelling individuals aged 18 years and over alive and resident in Northern Ireland (NI) who completed a 2011 Census return was followed up with linkage to mortality records until the end of December 2015. The data were accessed via the Administrative Data Research Centre Northern Ireland (ADRCNI) which provides fully anonymised datasets to accredited researchers within a secure data environment. The Strengthening the Reporting of Observational Studies in Epidemiology (STROBE) guidelines for reporting cohort studies was followed (von Elm *et al*., 2007) and a flowchart showing selection criteria is presented in Fig. 1. Institutionalised individuals and those younger than 18 years were excluded due to the rarity of the outcome in these subgroups.

### Measures of multimorbidity and activity limitation

The NI 2011 Census contains a rich array of health and morbidity measures. Participants were asked, ‘*Do you have any of the following conditions which have lasted, or are expected to last, at least 12 months*?’ People could tick all the conditions from a list of conditions that related to them. For this study, the following were considered as physical morbidities: (i) ‘*deafness or partial hearing loss*’; (ii) ‘*blindness or partial sight loss*’; (iii) ‘*communication difficulty (a difficulty with speaking or making yourself understood)*’; (iv) ‘*a mobility or dexterity difficulty (a condition that substantially limits one or more basic physical activities such as walking, climbing stairs, lifting or carrying)*’; (v) ‘*a learning difficulty, an intellectual difficulty, or a social or behavioural difficulty*’; (vi) ‘*long-term pain or discomfort*’; (vii) ‘*shortness of breath or difficulty breathing (such as asthma)*’; (viii) ‘*frequent periods of confusion or memory loss*’. However, responses to this question only register the presence or absence of morbidity and not the associated severity or effects. To address this limitation, the responses to a second question on activity limitation were included; this asked … *‘Are your day-to-day activities limited because of a health problem or disability which has lasted, or is expected to last, at least 12 months?*’ with responses, *no; yes-limited a little; yes-limited a lot*. Previous research has demonstrated that this is very closely related to physical aspects of health and especially to mobility issues (Wright *et al*., 2017).

### Measures of mental health

Mental health status was also based on the responses to the Census question that asked if people had experienced ‘*an emotional, psychological or mental health condition (such as depression or schizophrenia*)’ which had lasted, or was expected to last at least 12 months. This indicator has been used as a measure of mental ill-health in a range of previous studies (Tseliou *et al*., 2016; Bosqui *et al*., 2017; Doebler *et al*., 2017; Maguire *et al*., 2017; Wright *et al*., 2018; Rosato *et al*., 2019).

### Covariates

All individual, household and area-level cohort attributes were drawn from responses to the 2011 Census and selected on the basis of a known association to mental health or suicide risk. Thus religious affiliation (categorised as Protestant, Catholic and none/other) and whether the person was living in a single-person household were also added to the standard demographic characteristics of age and sex, and marital status (grouped as never married; married or co-habiting; separated, widowed, divorced) (Spoerri *et al*., 2010; O'Reilly and Rosato, 2015). Socioeconomic status was assessed from the Census responses on educational attainment (no formal qualifications; basic; A-levels; first degree or higher) and a combination of housing tenure and the capital value of the property. The capital value of all private houses in NI had been derived as part of an exercise in 2005 by the central government to determine the level of local tax payable by each household (O'Reilly *et al*., 2012). These data were combined with housing tenure from the Census to produce an eight-fold classification of tenure/capital value of property: private renting; social renting; and, for owner-occupiers, six categories ranging from less than £ 75 000 to over £ 200 000, with a separate category for owners with homes as yet unvalued. Housing tenure/value has been shown to be predictive of future mortality (Connolly *et al*., 2010). Two area-level indicators of the character of the area of residence were included. The first, an indicator of urban/rural residence was based on an official classification of settlements (Northern Ireland Statistics and Research Agency [NISRA], 2015) and was re-categorised as urban (comprising settlements of >75 000 people), intermediate (2500–75 000 people) and rural (<2500 people). The second, an indicator of area deprivation, was assigned using the Income domain of the NI Index of Multiple Deprivation which is based on the proportion of individuals on means-tested social security benefits (Devlin *et al*., 2018). Areas were defined as 890 homogenous groups of approximately 1900 individuals and were ranked from least deprived to most deprived, then split into equal quintiles.

### Mortality

Information on the month and year of death, as well as the primary cause of death recorded using ICD-10 codes, were extracted from the linked mortality records supplied by the General Register Office of Northern Ireland. Deaths were classified as ‘suicide’ and in accordance with established practice, using ICD-10 codes for both intentional self-harm (ICD10: X60–X84, Y87.0) and deaths of undetermined intent (ICD-10 code: Y10–Y34; Y87.0; Y87.2).

### Analytical strategy

Although multimorbidity is usually defined as the presence of two or more chronic conditions (WHO, 2016), we were keen to make use of all the data on physical health and to explore gradients according to the number of physical morbidities, so, for the most part the number of physical morbidities was categorised as 0, 1, 2, 3, 4 or more. Descriptive statistics recorded the demographic and socio-economic characteristics of the population at baseline along with the proportion of death by suicide. The relationship between physical morbidities, activity limitation and suicide was examined using separate logistic regression models, both before and after inclusion of chronic mental ill-health with progressive adjustment for other relevant demographic and socio-economic factors. It has been demonstrated that results from logistic regression and Cox regression models are similar when the follow-up period is short and the outcome is rare (Green and Symons, 1983; Annesi *et al*., 1989; Ingram and Kleinman, 1989; Callas *et al*., 1998). Tests for interaction were undertaken to determine if the relationship between physical health and suicide risk varied according to age. The analysis was stratified by age to determine whether physical ill-health was predictive of suicide risk at older ages. STATA software version 15 was used for all data analyses.

### Ethics approval

The resulting linked data were anonymised, held in the ADRCNI secure data environment within NISRA and made available to the research team for the purpose of this study. Approval for this study was obtained from the Office for Research Ethics Committees Northern Ireland (ORECNI), approval reference number 16/SC/0241. Informed consent from the subjects was not necessary because only anonymised data were released to the research team.

## Results

Table 1 describes the sociodemographic characteristics of the cohort. Overall, 7.4% reported chronic poor mental health and 13.7% reported two or more physical health conditions (i.e. multimorbidity); 7.7% had two physical conditions, 4.0% had three and 2.0% had more than or equal to four conditions. Overall, one-in-four (25.4%) reported that their daily activities were limited a little or a lot. The variation of the prevalence of multimorbidity and activity limitation across the sociodemographic strata of the cohort were as expected (see online Supplementary Table 1s). The prevalence of both indicators of physical ill-health increased markedly with age affecting almost half of those aged 75 years and over and was more common amongst those who were not currently married and those living in single-person households. Strong socioeconomic gradients were evident across all indicators – educational attainment, housing tenure/value, area or residence deprivation score. Multimorbidity and activity limitation was present in 42.2% and 75.9%, respectively, of those with self-reported poor mental health, and in 21.6% and 44.1% of those who died by suicide. Over the follow-up period, a total of 51 672 individuals died, of whom 877 were due to suicide. The distribution of suicide deaths across the sociodemographic characteristics of the population was similar to that of the physical health measures with the notable exception of age and sex, with death due to suicide more common at younger ages and three times more frequent amongst men compared to women (225 suicide deaths in women compared to 652 in men [see Table 1]).

**Table 1.** Socio-demographic characteristics of the Northern Ireland cohort aged 18 + years (*N* =  1 196 364) and proportion of death by suicide

	Study population (*N* = 1 196 364) *n* (%)	Death by suicide (*N* = 877) *n* (row %)
Multiple physical morbidity		
None	854 991 (71.5)	554 (0.06)
1 condition	176 900 (14.8)	134 (0.08)
2 conditions	92 068 (7.7)	97 (0.11)
3 conditions	48 167 (4.0)	56 (0.12)
4+ conditions	24 238 (2.0)	36 (0.15)
Limiting long-term illness		
None	892 124 (74.6)	490 (0.05)
A little	129 913 (10.9)	129 (0.10)
A lot	174 327 (14.6)	258 (0.15)
Self-reported poor mental health		
No	1 107 517 (92.6)	602 (0.05)
Yes	88 847 (7.4)	275 (0.3)
Gender		
Female	628 662 (52.6)	225 (0.04)
Male	567 702 (47.5)	652 (0.11)
Age (at 2011 Census), years		
18–24	138 596 (11.6)	119 (0.09)
25–34	205 415 (17.2)	167 (0.08)
35–44	222 155 (18.6)	197 (0.09)
45–54	224 269 (18.8)	206 (0.09)
55–64	176 641 (14.8)	117 (0.07)
65–74	131 891 (11.0)	51 (0.04)
75+	97 397 (8.1)	20 (0.02)
Marital status		
Never married	328 183 (27.4)	384 (0.12)
Married/cohabiting	678 233 (56.7)	311 (0.05)
Separated/divorced/widowed	189 948 (15.9)	182 (0.10)
Persons in household		
Others	989 599 (82.7)	571 (0.06)
Single-person household	206 765 (17.3)	306 (0.15)
Religion		
Protestant	533 409 (44.6)	326 (0.06)
Catholic	465 061 (38.9)	335 (0.07)
Other	197 894 (16.5)	216 (0.11)
Highest qualification		
No qualification	345 700 (28.9)	358 (0.10)
Basic education	409 044 (34.2)	337 (0.08)
A–levels	146 479 (12.2)	68 (0.05)
1st degree or higher	295 141 (24.7)	114 (0.04)
House tenure/value		
£ 200 000 +	160 424 (13.4)	57 (0.04)
£ 150 000–199 999	151 973 (12.7)	73 (0.05)
£ 100 000–149 999	272 801 (22.8)	130 (0.05)
£ 75 000–99 999	182 203 (15.2)	120 (0.07)
<£ 75 000	126 080 (10.5)	105 (0.08)
Private rent	161 191 (13.5)	169 (0.10)
Social rent	141 692 (11.8)	223 (0.16)
Area of residence		
Rural	322 067 (26.9)	168 (0.05)
Intermediate	637 013 (53.3)	483 (0.08)
Urban	237 284 (19.8)	226 (0.10)
Income deprivation index quintile		
1(least deprived)	233 484 (19.5)	111 (0.05)
2	253 058 (21.2)	150 (0.06)
3	246 545 (20.6)	144 (0.06)
4	241 478 (20.2)	188 (0.08)
5 (most deprived)	221 799 (18.5)	284 (0.13)

### Association between multimorbidity and mortality risk

Table 2 (full model results available on request) shows that most cases of suicide occurred in those with no reported physical health conditions or limitation of daily activity (554 and 490 cases, respectively). However, the risk of death from suicide increased directly and in proportion to the number of physical health conditions reported, and also to the severity of activity limitation even after adjustment for a wide range of sociodemographic and socioeconomic factors (models 1–3), though the gradients were more marked for activity limitation. The strongest relationship was with poor mental health (model 4) and people reporting poor mental health were almost four times as likely as their peers to die by suicide over the follow-up period (OR = 3.89; 95% CI 3.32–4.56). Model 5 shows that the association between the number of physical conditions and elevated suicide risk disappeared entirely with further adjustment for activity limitation, though the relationship between activity limitation and suicide risk remains relatively unchanged. The final model (model 6) shows that activity limitation remains a significant risk factor for suicide even after adjustment for poor mental health, with an increase of 57% and 72% for those whose activities are limited a little and a lot respectively (OR = 1.57; 95% CI 1.24–1.99 and OR = 1.72; 95% CI 1.35–2.20).

**Table 2.** Association between multimorbidity, limiting long-term illness and mental health status and death by suicide

Variable	Study population (*N* = 1 196 364) *n* (%)	Suicide (*N* = 877) *n* (row %)	Model 1 OR (95% CI)	Model 2 Adj. OR (95% CI)	Model 3 Adj. OR (95% CI)	Model 4 Adj. OR (95% CI)	Model 5 Adj. OR (95% CI)	Model 6 Adj. OR (95% CI)
Multiple physical morbidity								
None	854 991 (71.5)	554 (0.06)	Reference	Reference			Reference	Reference
1 condition	176 900 (14.8)	134 (0.08)	1.17 (0.97–1.41)	1.19 (0.98–1.45)	–	–	0.74 (0.60–0.92)	0.87 (0.70–1.07)
2 conditions	92 068 (7.7)	97 (0.11)	1.63 (1.31–2.02)	1.66 (1.32–2.09)	–	–	0.76 (0.58–1.00)	0.86 (0.66–1.12)
3 conditions	48 167 (4.0)	56 (0.12)	1.80 (1.36–2.36)	1.80 (1.35–2.40)	–	–	0.79 (0.57–1.09)	0.83 (0.60–1.14)
4 + conditions	24 238 (2.0)	36 (0.15)	2.29 (1.64–3.21)	1.97 (1.38–2.80)	–	–	0.86 (0.59–1.26)	0.76 (0.52–1.11)
Limiting long-term illness								
None	892 124 (74.6)	490 (0.05)	Reference	–	Reference	–	Reference	Reference
A little	129 913 (10.9)	129 (0.10)	1.81 (1.49–2.20)	–	2.11 (1.72–2.59)	–	2.43 (1.94–3.05)	1.57 (1.24–1.99)
A lot	174 327 (14.6)	258 (0.15)	2.70 (2.32–3.14)	–	2.64 (2.21–3.14)	–	3.13 (2.50–3.93)	1.72 (1.35–2.20)
Self-reported poor mental health								
No	1 107 517 (92.6)	602 (0.05)	Reference	–	–	Reference	–	Reference
Yes	88 847 (7.4)	275 (0.31)	5.71 (4.95–6.59)	–	–	3.89 (3.32–4.56)	–	3.12 (2.58–3.76)

OR, odds ratio; Adj. OR, adjusted odds ratio; CI, confidence interval.

Model 1 – unadjusted model.

Model 2 – multiple physical morbidities adjusted for sociodemographics (age, gender, marital status, single-person household, religion, highest qualification, house tenure/value, area of residence, income deprivation).

Model 3 – Limiting long-term illness (LLTI) adjusted for sociodemographics.

Model 4 – Mental health adjusted for sociodemographics.

Model 5 – adjustment for multiple physical morbidities, LLTI and sociodemographics.

Model 6 – adjustment for multiple physical morbidities, LLTI, mental health and sociodemographics.

Table 3 shows how the risk of suicide varies according to the degree of activity limitation stratified by the presence or absence of chronic poor mental health. Although most people with activity limitation did not report chronic poor mental health, there is a close correspondence between the degree of activity limitation and poor mental health which rises from 2.4% of those with no activity limitation to 27.3% of those with a lot of activity limitation. Again it is evident that about half of suicides (50.5%, *n* = 443/877) occurred in those with no activity limitation or poor mental health, though the risk of suicide increased with the degree of activity limitation, even in those without poor mental health (adjusted OR = 1.81; 95% CI 1.42–2.31 for those with a lot of limitation of daily activities, compared to their peers). However, the risk of suicide is much higher amongst people reporting poor mental health, either with or without activity limitation.

**Table 3.** Suicide risk according to the degree of activity limitation amongst those with and those without poor mental health

Poor Mental health	Activity limitation	Study population (*N* = 1 196 364) *n* (%)	Suicide (*N* = 877) *n* (row %)	Model 1 OR (95% CI)	Model 2 Adjusted OR (95% CI)
No	No limitation	870 691 (72.8)	443 (0.05)	Reference	Reference
No	Limited a little	110 091 (9.2)	62 (0.06)	1.11 (0.85–1.44)	1.42 (1.08–1.88)
No	Limited a lot	126 735 (10.6)	97 (0.08)	1.50 (1.21–1.87)	1.81 (1.42–2.31)
Yes	No limitation	21 433 (1.8)	47 (0.22)	4.32 (3.20–5.83)	3.94 (2.90–5.34)
Yes	Limited a little	19 822 (1.7)	67 (0.34)	6.66 (5.15–8.62)	4.99 (3.81–6.53)
Yes	Limited a lot	47 592 (4.0)	161 (0.34)	6.67 (5.57–7.99)	4.54 (3.70–5.56)

OR, odds ratio; CI, confidence interval.

Model 1 – unadjusted model.

Model 2 – adjusted for age, gender, marital status, single-person household, religion, highest qualification, house tenure/value, area of residence, area deprivation.

### Effect of age-group

**Fig. 2.**
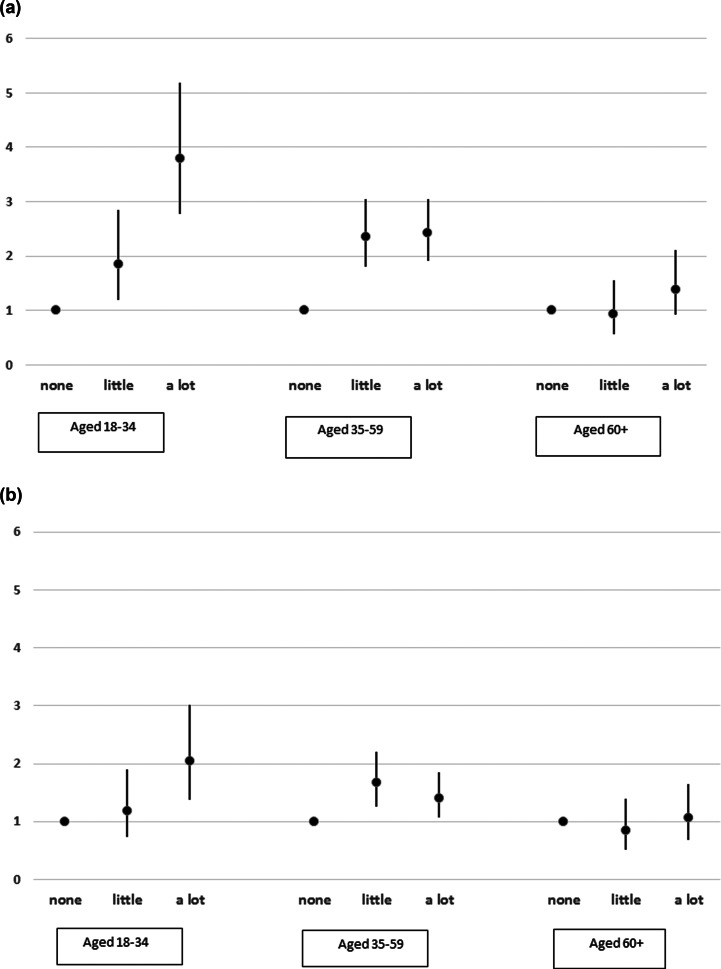
(*a*) Relationship between activity limitation and suicide risk stratified by age, before adjustment for mental health. Data represent odds ratios and 95% confidence interval from fully adjusted regression models. (*b*) Relationship between activity limitation and suicide risk stratified by age, after adjustment for mental health. Data represent odds ratios and 95% confidence interval from fully adjusted regression models.

Finally, further analysis was undertaken to determine if the relationship between physical health and suicide risk varied according to age. For this analysis, the cohort was divided into three age groups (18–34 years, 35–59 years, 60 years and over). Tests for interaction between age and activity were undertaken first in models adjusting for the sociodemographic and socioeconomic factors described in Table 1, and then with further adjustment for poor mental health. The number of physical conditions was not included in these models as the earlier analysis had demonstrated that this was not independently associated with suicide risk. Both interactions were significant (Likelihood ratio = 33.4, *p* < 0.001 and Likelihood ratio = 19.6, *p* = 0.001 respectively). Figure 2*a* summarises the relationship between suicide risk and degree of activity limitation stratified by age, before adjustment for poor mental health and full details are shown in the online Supplementary Table 2s. The effects of activity limitation were much more pronounced at younger ages, with an almost four-fold risk of suicide (OR = 3.79; 95% CI 2.77–5.19) for those aged 18–34 years reporting a lot of activity limitation compared to their peers with no activity limitation. This effect reduced to a two-fold risk (OR = 2.04; 95% CI 1.38–3.02) after adjustment for poor mental health (Fig. 2*b*). People aged 60 years and over with a lot of activity limitation were not at a significantly increased risk of suicide compared to their peers, either before or after adjustment for poor mental health (OR = 1.39; 95% CI 0.92–2.11 and OR = 1.07; 95% CI 0.69–1.65, respectively).

## Discussion

### Main findings

This population-wide study of over 1 million residents of NI examined the relationship between different aspects of physical health and suicide, and whether this relationship varied by age. It found an initial association between the number of physical health conditions and suicide risk. However, this association disappeared following adjustment for activity limitation suggesting that it is not the number of conditions but the effect of activity restriction that matters. There was a dose-response relationship between activity limitation and suicide risk which remained a significant risk factor even after adjusting for chronic poor mental health. The association was also evident amongst those who did not report poor mental health. The effect of activity limitation on suicide was more pronounced at younger ages (under 60 years). Adjustment for poor mental health attenuated the association between activity limitation and suicide risk in younger adults. There was no statistically significant elevation of suicide risk based on activity limitation in older adults (60 years and over) relative to their peers, either before or after adjustment for poor mental health.

### Comparison with other studies

Our study findings agree in part with earlier studies (Ahmedani *et al*., 2017; Wei and Mukamal, 2019; Zhuang *et al*., 2019) which demonstrated an association between the number of physical health problems and suicide risk, though we extended this by considering another dimension of physical health, activity limitation, which was absent in these studies. Our findings that the number of physical conditions is no longer associated with suicide risk after adjustment for the effect that they have on activities of daily living are in agreement with a population-based study conducted in the USA (Kaplan *et al*., 2007) though the authors did not consider variation by the degree of activity limitation and age group. Our finding of an association between activity limitation and suicide risk is in agreement with a study conducted in China (Cao *et al*., 2019) though the authors did not consider the number of physical illness and individuals younger than 60 years. A recent systematic review found evidence of an association between functional disability and suicidal behaviour (including death by suicide) in adults aged over 50 years (Lutz and Fiske, 2018), but our study suggests this association is more pronounced in younger age groups.

### Possible mechanisms

There could be several explanations for this relationship between activity limitation and suicide. Research suggests that individuals with disability face stigma, discrimination and a range of other social and economic challenges which compromise their mental wellbeing and increase their likelihood of having suicidal thoughts (Milner *et al*., 2019). Loss of autonomy, becoming a burden to carers, despondency arising from the inability to deal with chronic unremitting pain, and cognitive and functional declines of dementia have been reported as precipitants of suicide (Choi *et al*., 2019). Drawing upon the interpersonal theory of suicide, heightened feelings of burdensomeness, loneliness and social isolation among individuals with a disability could spiral to depression and trigger suicidal thoughts and attempts (Khazem, 2018). People with disabling conditions tend to have sleep disturbances (Kinne *et al*., 2004; Shandra *et al*., 2014) and findings from a systematic review revealed that sleep disturbances are independent risk factors for suicide (Bernert *et al*., 2015) This, however, might not be true in all situations – a large-scale study of 169 373 individuals in the USA found an association between physical health problem (restless leg syndrome) and suicide risk even after adjusting for the effect of sleep disturbance, depression and other covariates (Zhuang *et al*., 2019).

Counter to expectations, there was no indication that activity limitation or multimorbidity was associated with increased suicide risk at older ages. The reason for this is not clear and could probably reflect the inability of the health measures here to detect the rather unique severity of the health conditions required to trigger such occurrences. It could also be that the coding processes failed to register such deaths. On the other hand, we showed that activity limitation as a suicide risk factor was more significant in younger people, and especially in those aged less than 35 years. The almost four-fold risk (for those with a lot of limitation of daily activity) was reduced by a factor of two in the model that further adjusted for poor mental health. We interpret this as indicating that chronic poor mental health is on the causal pathway to suicide, i.e. most of the younger people with chronic activity limitation who take their lives do so because of the associated poor mental health. This suggests that perhaps a recognition and management of mental health in younger people with poor physical health may save lives. Another possible explanation for this variation by age group is role expectation. Since physical ill-health increases with age (Barnett *et al*., 2012), it is possible that older adults anticipate age-related physical health conditions that might limit their activities and as such, might be better at managing their own role expectations. However, the inability to carry out daily activities normally expected of a young person could possibly cause considerable psychological distress. A study conducted among undergraduate students in the USA (mean age 22.8 years) found that those with disability reported higher perceived burdensomeness (Khazem *et al*., 2015), which is known to be associated with suicidal behaviours.

It is likely that poor mental health may still be important as it is likely that people with activity limitation may suffer intermittent periods of poor mental health which would not have been recorded in this study. Taken together, our findings suggest that suicide prevention should target individuals experiencing activity limitation irrespective of their mental health status. Health professionals, carers and family members/friends or other support systems need to be aware of and lookout for signs of psychological distress in general and suicidal behaviours.

### Strengths and limitations

This study has several strengths. It is a population-wide study and does not just target specific subgroups. Physical and mental health were measured first and the large sample offered sufficient power to provide robust estimates for suicide rather than intermediate outcomes such as suicide ideation. Data were based on official statistics and therefore included validated cause of death. Although the linkage of administrative data records ensures data completeness and minimises losses to follow up, it is possible that some individuals emigrated during the follow-up period and some deaths by suicide might have been missed. However, the proportion of international migration is low and decreasing in Northern Ireland, estimated loss ranging from 12 500 in 2011 to 10 300 in 2015 (NISRA 2013; NISRA 2016). Our study population was predominantly white, consistent with evidence from the last two Censuses (NISRA 2013), which limits the generalisability of the findings to more heterogeneous populations. The measure of physical and mental health status was based on self-report and thus potentially subject to variability in perception and reporting, though we do not believe that this would have biased the association between activity limitation and suicide risk. Self-reported mental health might also have issues with face validity and predictive validity. On the other hand, any survey that would facilitate detailed assessment of physical health would lack the power to examine effects on suicide risk. Available evidence suggests that suicide risk might be related to the timing of onset and sequence of the physical versus the mental health condition (Qin *et al*., 2014; Bolton *et al*., 2015) but we were not able to adjust for these variables with the data available to us. Finally, changes in attributes between baseline and endpoint is a common problem for cohort studies and not easily overcome. In terms of health status, we would note that the Census questions included a requirement for chronicity i.e. that the conditions had lasted or were expected to last at least 12 months, so it is anticipated that there would have been relatively little resolution over 4 years of follow-up. However, it is more likely that a proportion of the healthy comparators would have developed ill-health as they have aged a little. Collectively these changes would have reduced the differences between the ‘healthy’ and ‘less healthy’ groups identified at baseline suggesting that the effect sizes in the current study are an underestimate.

### Policy implications

Our results suggest that better management of individuals (especially younger adults) with physical health conditions that limit activities of daily living may help to reduce suicide. Clinicians should be aware of this increased risk and may wish to screen such individuals for psychological distress and offer treatment for any identified mental health conditions. Poor mental health (for example depression) in people with a chronic illness is less frequently detected by clinicians treating physical diseases (Goldberg, 2010). Thus, detection and management of poor mental health in people with physical disorders would be necessary for enhancing their quality of life and suicide prevention. It would be beneficial to educate the patients, their carers or family on suicidal behaviours and provide useful information about support resources available in the community. Additional steps toward improving the health of people with functional limitations would include increasing access to supportive devices/techniques, improved access to health care services and reducing barriers to their participation in the society (Lollar, 2002). Measures to assist young people with activity limitation to manage their role expectations and possibly reduce feelings of burdensomeness may, therefore, be valuable in reducing the suicide risk associated with chronic disease especially in younger people.

## Supplementary material

For supplementary material accompanying this paper visit http://dx.doi.org/10.1017/S2045796020000529. click here to view supplementary material

## Data

The authors received permission to access the data used in this study, however, they are unable to share the data as they are not the data custodians
