# Supplementary material for: Does physical ill-health increase the risk of suicide? A census-based follow-up study of over 1 million people
Source: Epidemiol Psychiatr Sci. 2020 Jul 8;29:e140. doi: 10.1017/S2045796020000529 (PMC7372180; doi:10.1017/S2045796020000529)
Supplement: Supplementary file 1 [file S2045796020000529sup001.docx]

**Supplementary Table 1s. Socio-demographic characteristics of the Northern Ireland cohort aged 18+years**

**(N=1,196,364) and proportion of multimorbidity (≥2 physical health conditions) and activity limitation**

|  |  |  | **Activity limitation^ⱡ^** | | |
| --- | --- | --- | --- | --- | --- |
| **Variable** | **Study population**  **(N=1,196,364)**  **n (%)** | **Multimorbidity^¶^**  **(N=164,473)**  **n (row %)** | **None**  **(N=892,124)**  **n (row %)** | **A little**  **(N=129,913)**  **n (row %)** | **A lot**  **(N=174,327)**  **n (row %)** |
| **Multiple physical morbidity** |  |  |  |  |  |
| None | 854,991 (71.5) | - | 797,300 (93.3) | 38,336 (4.5) | 19,355 (2.3 ) |
| 1 condition | 176,900 (14.8) | - | 82,664 (46.7) | 54,789 (31.0) | 39,447 (22.3) |
| 2 conditions | 92,068 (7.7) | - | 9,376 (10.2) | 26,251 (28.5) | 56,441 (61.3) |
| 3 conditions | 48,167 (4.0) | - | 2,003 (4.2) | 8,308 (17.3) | 37,856 (78.6) |
| 4+ conditions | 24,238 (2.0) | - | 781 (3.2) | 2,229 (9.2) | 21,228 (87.6) |
| **Limiting long-term illness** |  |  |  |  |  |
| None | 892,124 (74.6) | 12,160 (1.36) | - | - | - |
| A little | 129,913 (10.9) | 36,788 (28.3) | - | - | - |
| A lot | 174,327 (14.6) | 115,525 (66.3) | - | - | - |
| **Self-reported poor mental health** |  |  |  |  |  |
| No | 1,107,517 (92.6) | 126,954 (11.5) | 870,691 (78.6) | 110,091 (9.9) | 126,735 (11.4) |
| Yes | 88,847 (7.4) | 37,519 (42.2) | 21,433 (24.1) | 19,822 (22.3) | 47,592 (53.6) |
| **Gender** |  |  |  |  |  |
| Female | 628,662 (52.6) | 91,570 (14.6) | 461,039 (73.3) | 72,124 (11.5) | 95,499 (15.2) |
| Male | 567,702 (47.5) | 72,903 (12.8) | 431,085 (75.9) | 57,789 (10.2) | 78,828 (13.9) |
| **Age (at 2011 Census)*,*years** |  |  |  |  |  |
| 18 - 24 | 138,596 (11.6) | 3,555 (2.6) | 128,711 (92.9) | 5,485 (4.0) | 4,400 (3.2) |
| 25 - 34 | 205,415 (17.2) | 6,524 (3.2) | 187,623 (91.3) | 8,962 (4.4) | 8,830 (4.3) |
| 35 - 44 | 222,155 (18.6) | 14,183 (6.4) | 189,998 (85.5) | 14,429 (6.5) | 17,728 (8.0) |
| 45 - 54 | 224,269 (18.8) | 26,362 (11.8) | 173,636 (77.4) | 20,980 (9.4) | 29,653 (13.2) |
| 55 - 64 | 176,641 (14.8) | 34,682 (19.6) | 115,325 (65.3) | 24,700 (14.0) | 36,616 (20.7) |
| 65 - 74 | 131,891 (11.0) | 36,320 (27.5) | 68,604 (52.0) | 27,570 (20.9) | 35,717 (27.1) |
| 75+ | 97,397 (8.1) | 42,847 (44.0) | 28,227 (29.0) | 27,787 (28.5) | 41,383 (42.5) |
| **Marital status** |  |  |  |  |  |
| Never married | 328,183 (27.4) | 25,474 (7.8) | 270,961 (82.6) | 25,019 (7.6) | 32,203 (9.8) |
| Married/cohabiting | 678,233 (56.7) | 82,546 (12.2) | 524,550 (77.3) | 69,764 (10.3) | 83,919 (12.4) |
| Separated/divorced/widowed | 189,948 (15.9) | 56,453 (29.7) | 96,613 (50.9) | 35,130 (18.5) | 58,205 (30.6) |
| **Persons in household** |  |  |  |  |  |
| Others | 989,599 (82.7) | 111,772 (11.3) | 777,844 (78.6) | 93,695 (9.5) | 118,060 (11.9) |
| Single-person household | 206,765 (17.3) | 52,701 (25.5) | 114,280 (55.3) | 36,218 (17.5) | 56,267 (27.2) |
| **Religion** |  |  |  |  |  |
| Protestant | 533,409 (44.6) | 80,268 (15.1) | 388,526 (72.8) | 65,357 (12.3) | 79,526 (14.9) |
| Catholic | 465,061 (38.9) | 62,475 (13.4) | 348,058 (74.8) | 45,466 (9.8) | 71,537 (15.4) |
| Other | 197,894 (16.5) | 21,730 (11.0) | 155,540 (78.6) | 19,090 (9.7) | 23,264 (11.8) |
| **Highest qualification** |  |  |  |  |  |
| No qualification | 345,700 (28.9) | 98,316 (28.4) | 176,223 (51.0) | 60,270 (17.4) | 109,207 (31.6) |
| Basic education | 409,044 (34.2) | 41,063 (10.0) | 327,763 (80.1) | 39,221 (9.6) | 42,060 (10.3) |
| A–levels | 146,479 (12.2) | 7,935 (5.4) | 129,153 (88.2) | 9,468 (6.5) | 7,858 (5.4) |
| 1^st^ degree or higher | 295,141 (24.7) | 17,159 (5.8) | 258,985 (87.8) | 20,954 (7.1) | 15,202 (5.2) |
| **House tenure/value** |  |  |  |  |  |
| £200,000+ | 160,424 (13.4) | 11,769 (7.3) | 136,405 (85.0) | 13,051 (8.1) | 10,968 (6.8) |
| £150,000–199,999 | 151,973 (12.7) | 13,649 (9.0) | 124,926 (82.2) | 14,126 (9.3) | 12,921 (8.5) |
| £100,000–149,999 | 272,801 (22.8) | 32,004 (11.7) | 211,890 (77.7) | 28,786 (10.6) | 32,125 (11.8) |
| £75,000–99,999 | 182,203 (15.2) | 25,987 (14.3) | 134,325 (73.7) | 20,571 (11.3) | 27,307 (15.0) |
| < £75,000 | 126,080 (10.5) | 21,197 (16.8) | 88,234 (70.0) | 15,385 (12.2) | 22,461 (17.8) |
| Private rent | 161,191 (13.5) | 21,456 (13.3) | 120,151 (74.5) | 16,349 (10.1) | 24,691 (15.3) |
| Social rent | 141,692 (11.8) | 38,411 (27.1) | 76,193 (53.8) | 21,645 (15.3) | 43,854 (31.0) |
| **Area of residence** |  |  |  |  |  |
| Rural | 322,067 (26.9) | 37,113 (11.5) | 248,498 (77.2) | 33,326 (10.4) | 40,243 (12.5) |
| Intermediate | 637,013 (53.3) | 87,795 (13.8) | 475,625 (74.7) | 70,405 (11.1) | 90,983 (14.3) |
| Urban | 237,284 (19.8) | 39,565 (16.7) | 168,001 (70.8) | 26,182 (11.0) | 43,101 (18.2) |
| **Income deprivation index quintile** |  |  |  |  |  |
| 1(least deprived) | 233,484 (19.5) | 23,096 (9.9) | 189,472 (81.2) | 22,960 (9.8) | 21,052 (9.0) |
| 2 | 253,058 (21.2) | 29,295 (11.6) | 197,322 (78.0) | 26,225 (10.4) | 29,511 (11.7) |
| 3 | 246,545 (20.6) | 31,770 (12.9) | 186,103 (75.5) | 26,671 (10.8) | 33,771 (13.7) |
| 4 | 241,478 (20.2) | 37,234 (15.4) | 172,736 (71.5) | 28,031 (11.6) | 40,711 (16.9) |
| 5 (most deprived) | 221,799 (18.5) | 43,078 (19.4) | 146,491 (66.1) | 26,026 (11.7) | 49,282 (22.2) |

^¶^Multimorbidity – two or more physical health conditions. ^ⱡ^Due to a limiting long-term illness (LLTI)

**Supplementary Table 2s. Association between limiting long-term illness and mental health status and death by suicide stratified by age groups**

| **Variable** | **18-34 years old (N=344,011)**  **n (%)** | **Suicide**  **(N=286)**  **n (row %)** | **Model 1**  **OR (95% CI)** | **Model 2**  **AOR (95% CI)** | **Model 3**  **AOR (95% CI)** | **Model 4**  **AOR (95% CI)** |
| --- | --- | --- | --- | --- | --- | --- |
| **Limiting long-term illness** |  |  |  |  |  |  |
| None | 316,334 (92.0) | 201 (0.06) | Reference | Reference | - | Reference |
| A little | 14,447 (4.2) | 24 (0.17) | 2.62 (1.71–4.00) | 1.85 (1.20–2.85) | - | 1.19 (0.74–1.90) |
| A lot | 13,230 (3.9) | 61 (0.46) | 7.29 (5.47–9.71) | 3.79 (2.77–5.19) | - | 2.04 (1.38–3.02) |
| **Self-reported poor mental health** |  |  |  |  |  |  |
| No | 327,328 (95.2) | 213 (0.07) | Reference | - | Reference | Reference |
| Yes | 16,683 (4.9) | 73 (0.44) | 6.75 (5.17–8.81) | - | 4.50 (3.36–6.02) | 3.18 (2.20–4.60) |
|  |  |  |  |  |  |  |
|  |  |  |  |  |  |  |
|  |  |  |  |  |  |  |
| **Variable** | **35-59 years old**  **(N=536,965)**  **n (%)** | **Suicide**  **(N=468)**  **n (row %)** | **Model 1**  **OR (95% CI)** | **Model 2**  **AOR (95% CI)** | **Model 3**  **AOR (95% CI)** | **Model 4**  **AOR (95% CI)** |
| **Limiting long-term illness** |  |  |  |  |  |  |
| None | 425,783 (79.3) | 239 (0.06) | Reference | Reference | - | Reference |
| A little | 46,733 (8.7) | 82 (0.18) | 3.13 (2.43–4.02) | 2.35 (1.81–3.04) | - | 1.67 (1.26–2.20) |
| A lot | 64,449 (12.0) | 147 (0.23) | 4.07 (3.31–5.00) | 2.42 (1.92–3.04) | - | 1.41 (1.08–1.85) |
| **Self-reported poor mental health** |  |  |  |  |  |  |
| No | 483,525 (90.1) | 293 (0.06) | Reference | - | Reference | Reference |
| Yes | 53,440 (10.0) | 175 (0.33) | 5.42 (4.49–6.54) | - | 3.64 (2.96–4.48) | 2.97 (2.33–3.79) |
|  |  |  |  |  |  |  |
|  |  |  |  |  |  |  |
|  |  |  |  |  |  |  |
| **Variable** | **60-90+ years**  **(N=315,388)**  **n (%)** | **Suicide**  **(N=123)**  **n (row %)** | **Model 1**  **OR (95% CI)** | **Model 2**  **AOR (95% CI)** | **Model 3**  **AOR (95% CI)** | **Model 4**  **AOR (95% CI)** |
| **Limiting long-term illness** |  |  |  |  |  |  |
| None | 150,007 (47.6) | 50 (0.03) | Reference | Reference | - | Reference |
| A little | 68,733 (21.8) | 23 (0.03) | 1.00 (0.61–1.65) | 0.94 (0.57–1.55) | - | 0.84 (0.51–1.39) |
| A lot | 96,648 (30.6) | 50 (0.05) | 1.55 (1.05–2.30) | 1.39 (0.92–2.11) | - | 1.07 (0.69–1.65) |
| **Self-reported poor mental health** |  |  |  |  |  |  |
| No | 296,664 (94.1) | 96 (0.03) | Reference | - | Reference | Reference |
| Yes | 18,724 (5.9) | 27 (0.14) | 4.46 (2.91–6.84) | - | 4.10 (2.64–6.35) | 4.00 (2.52–6.34) |

OR – odds ratio. AOR – adjusted odds ratio.

Model 1 - unadjusted model

Model 2 – LLTI adjusted for sociodemographics ( gender, marital status, single-person household, religion, highest qualification, house tenure/value, area of residence, income deprivation)

Model 3 – Mental health adjusted for sociodemographics

Model 4 – adjusted for LLTI, mental health and sociodemographics
